# Diffuse Large B-Cell Lymphoma – Rare Presentation

**DOI:** 10.5334/jbsr.2747

**Published:** 2022-04-27

**Authors:** Rodrigo Cordeiro, Mariana Chaves

**Affiliations:** 1 Hospital do Divino Espírito Santo de Ponta Delgada, PT

**Keywords:** B-cell lymphoma, diffuse large B-cell lymphoma, subcutaneous tissue, CT, ultrasound

## Abstract

**Teaching Point:** Subcutaneous nodules sparing the dermis and without extracutaneous involvement is a rare presentation of the diffuse large B-cell lymphoma.

## Case History

**Figure 1:**
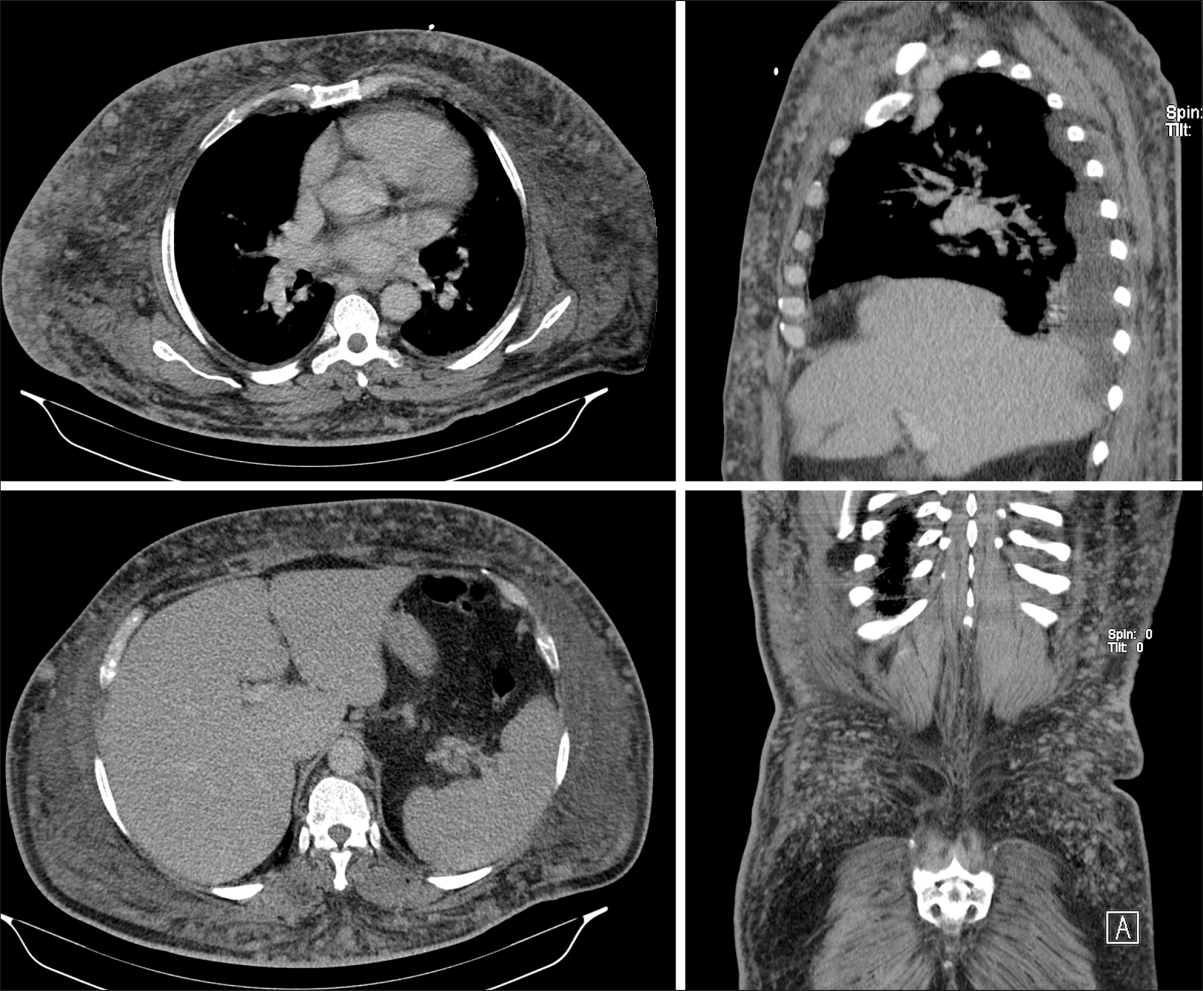


**Figure 2:**
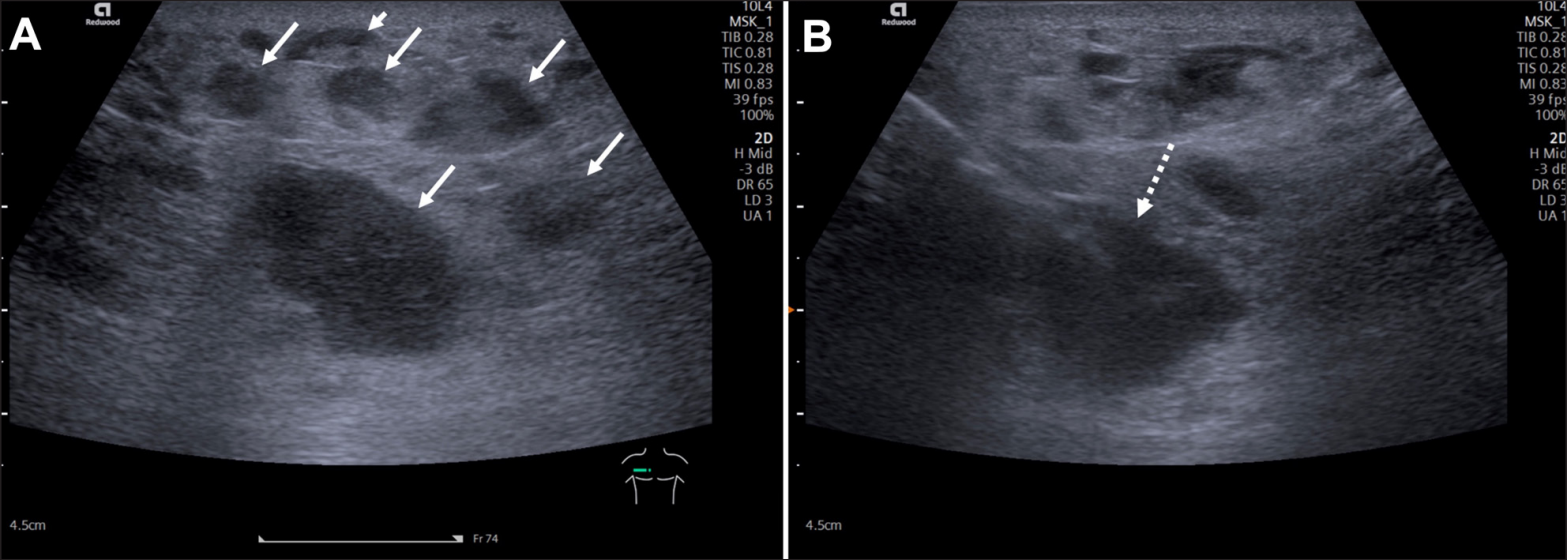


A 63-year-old male presented with fever, sweating, and anorexia with a few weeks’ duration. Blood tests revealed leukocytosis and high C-Reactive protein. A thoracoabdominal-pelvic computed tomography (CT) revealed multiple enhancing subcutaneous nodules spread throughout the abdominal and chest wall (***Figure 1***). CT didn’t show other relevant findings, namely adenopathies. Ultrasound study showed multiple solid subcutaneous nodules (***Figure 2A***), and an ultrasound-guided biopsy of one of these lesions was performed (***Figure 2B***). Additionally, two inguinal lymph nodes without suspicious sonographic criteria were biopsied. The lesion’s histology revealed a diffuse large B-cell lymphoma (DLBCL) confined in subcutaneous tissue without dermis’s invasion. The biopsy of the lymph nodes was negative. The dermatological evaluation revealed only the perception of generalized skin hardening. The immunophenotypic study of peripheral blood lymphocytes and the bone marrow didn’t show significant changes.

The patient underwent chemotherapy with R-CHOP, after which he was clinically well, with complete resolution of lesions on follow-up CT.

## Comment

DLBCL is the most common type of lymphoma; under the current World Health Organization (WHO) classification, about 80% are classified as not otherwise specified, and only 20% are considered specific variants.

Primary cutaneous lymphomas are restricted to the skin at the time of diagnosis and are rare manifestations of extranodal lymphomas, in which primary cutaneous B-cell lymphoma accounts for 20–25% of cases [1].

Primary cutaneous diffuse large B-cell lymphoma, leg type (PCDLBCL-LT) is the least common subtype of primary cutaneous B-cell lymphoma, characteristically more aggressive, and worse prognosis. It frequently affects elderly patients, predominantly female, manifesting as nodular lesions and/or rapidly growing erythematous-cyanotic plaques, classically in the leg, but may involve other sites in 15–20% of patients. B symptoms (fever, night sweats, and weight loss) may also appear [1]. Ultrasound may show cutaneous and subcutaneous thickening with locally organized nodules or plaques. CT can allow delineation of the nodule if it is large enough, but multiple subcutaneous nodules are more characteristic of subcutaneous panniculitis-like T-cell lymphoma. Diagnosis requires a biopsy that should include dermis and subcutaneous fat, complemented with appropriate staging to exclude systemic disease [1].

This case shows a rare presentation of DLBCL with a predominance of B symptoms, without visible cutaneous manifestations, and widespread involvement of the subcutaneous fat. According to the current WHO classification, the histological findings didn’t allow us to diagnose as PCDLBCL-LT, so the diagnosis of DLBCL not otherwise specified was assumed. There are few cases described of DLBCL restricted to the subcutaneous fat, sparing the dermis and without extracutaneous involvement.

This case reinforces the importance of evaluating the subcutaneous tissue as an integral part of the radiological reading.
